# Mesenchymal Stem Cell-Derived Extracellular Vesicles: Pleiotropic Impacts on Breast Cancer Occurrence, Development, and Therapy

**DOI:** 10.3390/ijms23062927

**Published:** 2022-03-08

**Authors:** Yiling Guo, Yujia Zhai, Longyuan Wu, Yazhuo Wang, Puzhen Wu, Lixia Xiong

**Affiliations:** 1 Department of Pathophysiology, Medical College, Nanchang University, 461 Bayi Road, Nanchang 330006, China; gyl2360100168@163.com (Y.G.); jp4217118118@qmul.ac.uk (Y.Z.); 6300817039@email.ncu.edu.cn (L.W.); 2 Second Clinical Medical College, Nanchang University, Nanchang 330006, China; 3 Queen Mary School, Nanchang University, Nanchang 330006, China; 4 First Clinical Medical College, Nanchang University, Nanchang 330006, China; 5 Medical College of Rehabilitation, Southern Medical University, Guangzhou 510515, China; wangyazhuo2000@outlook.com; 6 Beijing-Dublin International College, Beijing University of Technology, Beijing 100124, China; puzhen.wu@ucdconnect.ie; 7 Key Laboratory of Functional and Clinical Translational Medicine, Xiamen Medical College, Fujian Province University, Xiamen 361023, China

**Keywords:** breast cancer, mesenchymal stem cell-derived extracellular vesicles, progression, therapeutic strategies

## Abstract

Breast cancer (BC) is one of the most devastating cancers, with high morbidity and mortality, among the female population worldwide. In BC, mesenchymal stem cells (MSCs), as pluripotent stromal stem cells, play a significant role in TME formation and tumor progression. Recently, an increasing number of studies have demonstrated that extracellular vesicles (EVs) are essential for the crosstalk between MSCs and BC cells. MSC-derived EVs (MSC-EVs) can deliver a diversity of molecules, including lipids, proteins, and nucleic acids, etc., to target cells, and produce corresponding effects. Studies have demonstrated that MSC-EVs exert both inhibitory and promotive effects in different situations and different stages of BC. Meanwhile, MSC-EVs provide novel therapeutic options for BC, such as EVs as carriers for drug delivery. Therefore, in this review, we summarize the role of MSC-EVs in BC progression and application in clinical treatment, in the hope of providing a basis for further research.

## 1. Introduction

Breast cancer (BC), among the most common malignancies in women worldwide, is characterized by the highest incidence, high recurrence rate, mortality, and poor prognosis [1,2,3]. BC progression is associated with multiple steps, such as proliferation, apoptosis, autophagy, invasion, migration, metastasis, angiogenesis, immune regulation, and dormancy. Based on molecular categories (estrogen receptor (ER), progesterone receptor (PR), and human epidermal growth factor receptor-2 (HER-2)), BC can be divided into luminal A, luminal B, normal breast-like, HER-2 over-expressing, and basal-like type [4]. Triple negative BC (TNBC) is a subtype of basal-like BC without expressing ER, HER-2, and PR on the surface of cancerous cells [5,6,7]. The properties of large tumor size, high proliferative capacity with poor differentiation, and lack of recognized biomarkers make conventional treatment for TNBC patients ineffective with a high recurrence rate [8,9]. Although surgery, chemotherapy, and radiotherapy have made significant progress in BC treatment in recent years, BC remains one of the most common causes of female death [10]. Therefore, it is necessary to explore the deeper mechanisms of cancer progression and illustrate new treatments that are more effective for BC.

**Figure 1:**
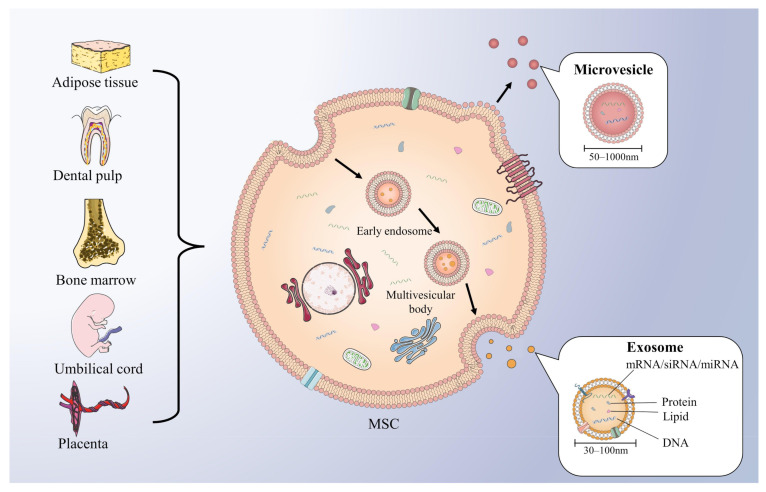
Various sources of MSCs and the generation of EVs. MSCs are mainly originated from adipose tissue, dental pulp, bone marrow, umbilical cord, and placenta. Inward budding formed early endosomes matured to form multivesicular bodies, which fuse with plasma membrane release exosomes. Exosomes (30–150 nm) are EVs contains miRNA, protein, lipid, and DNA. Microvesicles (50–1000 nm) are EVs released directly from the plasma membrane.

Extracellular vesicles (EVs) are a heterogeneous group of membrane-bound vesicles that can be released from their original cells by invagination and budding. EVs then facilitate cell-to-cell interactions via contact with neighbor cells or internalization by recipient cells, which includes fusion with membrane and endocytosis [11]. Based on the biogenesis, biophysical properties, and function, EVs can be classified into three main subtypes, including exosomes, microvesicles (MVs), and apoptotic blebs [12]. (1) Exosomes, with a diameter of 30–150 nm, originate from vesicles formed by the inward budding of the limiting membrane of early endosomes (EEs), also known as intraluminal vesicles (ILVs). When the EEs mature into multivesicular bodies (MVBs), they can fuse with the plasma membrane and release enclosed ILVs in the extracellular space, at which point the ILVs are called exosomes [13]. (2) MVs are vesicles 50–1000 nm in diameter that bud directly from the plasma membrane [14]. (3) Apoptotic blebs, 1000–5000 nm in diameter, are EVs released during programmed cell death and contain intact organelles, DNA, and histones [15]. This review focuses on two main subtypes of EVs involved in BC: exosomes and MVs, which play important roles in intercellular communication, antigen presentation, and tumor progression [12]. Both of them contain lipids, proteins, and genetic material, such as DNA, functional messenger RNA (mRNA), microRNA (miRNA), and small interfering RNA (siRNA) (Figure 1) that can be horizontally delivered to recipient cells and reprogrammed [16]. As the distinction between “exosomes” and “MVs“ is ambiguous, the two concepts have been used interchangeably in many published studies [14]. Recent studies have demonstrated that EVs are inextricably linked to the progression and treatment of cancer. Through EV secretion, both tumor metastasis and angiogenesis can be increased by transferring oncogenic growth factor receptor and their ligand [17,18]. Moreover, via the transfer of various tumor-related proteins, EVs can also promote the growth and drug resistance of tumors [19,20]. Considering that exosomes with good biocompatibility can be specifically modified and deliver cargoes to the targeted cells, clinically, they are designed to deliver various therapeutic payloads, such as miRNAs, peptides, and synthetic drugs [21]. Therefore, targeting exosomal molecules and exosome-based drug delivery systems may bring about a great revolution in cancer treatment. However, finding the appropriate exosomal molecules and donor cells to establish an effective exosomal drug delivery system needs further research. 

Exosome-mediated cell-to-cell crosstalk between tumor cells and the stroma and immune cells of the tumor microenvironment (TME) is critical to the pathophysiology of cancer [22,23]. For instance, EVs can modulating the TME of glioblastoma tumor into a tumor-promoting system to promote tumor establishment [24]; also, EVs can communicate with the microenvironment in gastric cancer to regulate the progression of cancer [25]. As a major component of the TME, mesenchymal stem cells (MSCs) are multipotent stromal stem cells found within most cancers and play an essential role in influencing TME formation and tumor progression [23]. In many cancers, such as breast, ovarian, lung and colon carcinomas, the MSCs can be recruited to TME by tumor-homing functions [26,27,28]. Through tumor homing to TME, interaction with immune cells and further polarization in TME, MSCs obtain the ability to play a dynamic role in different types of cancer [29].

According to different tissue sources, MSCs are mainly classified into bone marrow-derived MSCs (BMSCs), adipose tissue-derived MSCs (AT-MSCs), umbilical cord-derived MSCs (UC-MSCs), dental pulp-derived MSCs (DPSC), and placenta-derived MSCs [30] (Figure 1). They exhibit a high capacity for self-renewal and multipotent differentiation. As multipotent cells, MSCs can be recruited to sites of injury and inflammation for tissue repair [31]. Similar to the site of injury, the tumor site has been likened to a “wound that never heals”, meaning that MSCs are also able to migrate to the tumor stoma as a key element of TME and contribute to tumor progression [32,33,34]. MSCs have been recently described to localize to BC, where they integrate into the tumor-associated stroma [34]. It is reported that multiple chemokines, cytokines, and growth factors, such as monocyte chemotactic protein-1 (MCP-1), cyclophilin B, hepatoma-derived growth factor (HDGF), urokinase plasminogen activator (uPA), and interleukin (IL)-6, can promote MSCs recruitment to BC cells. Then, MSCs appear to promote tumor formation and progression either by acting directly on BC cells or indirectly on TME [35]. On the one hand, MSCs regulate tumor progression through juxtacrine, paracrine, and endocrine mechanisms, directly secreting a variety of bioactive molecules and paracrine factors, which promote the tumorigenic ability of BC cells by regulating TME under the mechanisms of immune regulation and connective tissue proliferation [34,35,36]. On the other hand, MSCs modulate tumor progression through the indirect secretion of EVs. 

In the TME, MSCs interact with the tumor by making and releasing EVs to horizontally transfer information to neighboring cells, to shift the TME into a tumor supportive or inhibitory milieu for tumor survival or suppression [37,38]. Because of the nature of EVs to reflect the characteristics of the parent cells, MSC-derived EVs (MSC-EVs) (MSC-derived exosomes (MSC-exos) and MSC-derived MVs (MSC-MVs)) exhibit a vital role in mediating intercellular communication at different stages of BC, and differences may exist in EVs derived from MSCs of different phenotypes. MSCs have the advantages of easy isolation and strong expansion ability in vitro and can produce a large number of more stable EVs with regenerative characteristics. In contrast to embryonic stem cells (ESCs), MSCs are free from legal and ethical considerations [39] as well as teratoma-generation concerns [40,41]. Therefore, research on MSCs is of more experimental significance and clinical application value. In this review, we will focus on the functions and regulatory mechanisms of MSC-EVs in BC progression and how to utilize MSC-EVs for better anti-BC therapy. 

## 2. Mesenchymal Stem Cell-Derived Extracellular Vesicles in Breast Cancer Occurrence and Development

Due to the interactions between cancer cells and multiple resident cells, intercellular communication in the tumor environment becomes very complex, and EVs are considered to be the main factors for cell-to-cell communication in TME [42]. Accumulated evidence suggests that MSC-EVs can deliver cargoes, such as proteins, mRNA, and miRNA to recipient cells, and thus exert various effects on the progression and drug response of different cancer cells [43]. 

In lung cancer and prostate cancer, the progression of the tumor can both be suppressed under the effect of MSC-EVs [44,45,46]. The tumorigenesis of colorectal cancer and bladder cancer can be restrained by regulating the c-MYC/DNA methyltransferase 3a (DNMT3a)/phosphatase and tensin homolog deleted on chromosome 10 (PTEN) axis and targeting polycomb repressor complex 1 (PRC1), respectively [47,48]. MSC-EVs loaded with miR-193a downregulated focal adhesion kinase (FAK) to inhibit colon cancer cell proliferation, migration, and invasion [49]. For tumor growth, MSC-EVs showed pro-tumor effects in lung and gastric cancers and anti-tumor function on liver cancer [50,51,52]. In hepatocellular carcinoma, MSC-EVs carrying miRNA-20a-3p can target cFLIP to promote tumor apoptosis [53]. In addition, in gastric cancer, tumor angiogenesis and metastasis were suppressed by miR-6785-5p encapsulated in MSC-EVs [54]. In colorectal cancer, the MSC-EVs exerted dual effects on immune evasion via different cargos and different mechanisms [55,56].

Therefore, understanding MSC-EV-mediated mechanisms between cancer cells and their microenvironment in cancer progression is of great significance for exploring new approaches to cancer treatment. In this section, we describe the exact roles of MSC-EVs in cell proliferation, autophagy, apoptosis, aggressiveness, angiogenesis, immune regulation, dormancy, and drug resistance of BC.

### 2.1. Cell Proliferation

Cell proliferation is a complex process that mainly contributes to an increase in the number of cells. For normal cells, cell proliferation is under dynamic equilibrium, however, some human MSCs (hMSCs) break this balance in BC cells by secreting EVs. Different signaling pathways transmit signals from the cell surface to the nucleus through a variety of mechanisms, and dysregulation of those signaling pathways that are closely linked to development may lead to tumorigenesis. In support of this view, Zhou et al. demonstrated that the activation of the extracellular signal-regulated kinase (ERK) signaling pathway by hUC-MSC-EVs can promote BC cell proliferation in vitro. In addition, Western blot analysis of epithelial–mesenchymal transition (EMT) relevant markers demonstrated that the expression of N-cadherin increased and E-cadherin decreased in hUC-MSC-EVs-treated BC cells, and the opposite results were obtained when ERK inhibitor was added. Together, these data suggest that hUC-MSC-EVs promote the proliferation of BC cells by activating the ERK pathway to induce EMT [57]. ERK is a member of the mitogen-activated protein kinase (MAPK) family, which plays a key role in cell proliferation and survival [58]. In addition, Wnt signaling can be also involved in tumor development, which is characterized by nuclear accumulation of β-catenin [59]. Studies have demonstrated that after exosomes derived from AT-MSCs (AT-MSC-exos) treatment, the proliferation of MCF7 cells was enhanced, and the expression levels of β-catenin and Wnt target genes, such as Axin2 and Dkk1, are increased. All these results indicated that AT-MSC-exos can activate the Wnt signaling pathway and further promote BC cell proliferation [60]. Vallabhaneni et al. isolated EVs from serum-deprived hMSCs and characterized their secreted cargoes. It revealed that hMSC-EVs could transport tumor-regulating miRNA, proteins, and metabolites, most of which can promote BC cell proliferation [61]. For example, miR224-5p extracted from hUC-MSC-exos were able to promote BC cell proliferation in vivo and in vitro [62]. In addition, in TNBC, the secretion of miR-106a-5p loaded BMSC-exos was suppressed by long non-coding RNA heart and neural crest derivatives expressed 2-antisense RNA 1 (lncRNA HAND2-AS1), which then has an inhibitory effect on tumor proliferation [63]. Therefore, we speculate that MSC-exosomal-miR-106a-5p may support TNBC growth.

hMSC-EVs can not only break the equilibrium, but also stabilize it. Mirabdollahi et al. demonstrated that EVs derived from one kind of hUC-MSCs called human Wharton’s jelly MSCs (hWJMSCs) can inhibit the proliferation of MCF-7 and 4T1 tumor cells in vitro. In vivo, tumor incidence, size, and weight were all reduced with the intervention of hWJMSC-EVs therapy [64]. Likewise, hUC-MSC-exos with over-expressed miR-148b-3p exerted an inhibitory effect on MDA-MB-231 cell proliferation [65]. 

Above all, MSC-EVs can abnormally re-awake development-related signaling pathways or transport tumor supporters to augment BC cells proliferation. Meanwhile, tumor suppressors can also be transported by MSC-EVs to oppositely demonstrate an inhibitory effect on the proliferation of BC cells.

### 2.2. Cell Autophagy and Apoptosis

Apoptosis is a type of programmed cell death removal of damaged or senescent cells and is characterized by morphological and biochemical changes in cells [66]; this process is regulated by the extrinsic and/or intrinsic signaling pathways and has a significant role in normal development and homeostasis [67]. Autophagy is considered a catabolic process that transfers cytoplasmic proteins or organelles encapsulated in autophagosomes to lysosomes for degradation or renewal, thereby maintaining natural cell growth and cellular homeostasis [68]. It is reported that autophagy contributed to the removal of damaged cellular compartments and recycling the components, thereby preventing apoptosis and improving survival [69]. A study demonstrated that miR-224-5p carried by hUC-MSC-exos can promote autophagy of BC cells by inhibiting its target gene HOXA5, thus suppressing the apoptosis of BC cells [62], which suggested a promoting role of MSC-EVs in BC. However, MSC-EVs can also induce apoptosis to produce anticancer effects. It is reported that AT-MSCs-derived MVs loaded with apoptosis-induced factors and substances can induce apoptosis and inhibit the proliferation of BC cells by upregulating Bax, P53, E2F2, and SMAD5 genes and downregulating the bcl gene [70]. Similarly, hUC-MSC-exosomal miR-148b-3p is able to enhance MDA-MB-231 cell apoptosis [65]. In addition, a study co-cultured hWJMSCs with BC cells and demonstrated that EVs of MSCs have a cytotoxic effect against BC cells by inducing apoptosis [71]. 

In general, the role of MSC-EVs in the autophagy and apoptosis of BC cells is complex, and its specific mechanism contributing to the dual functions needs to be further studied. 

### 2.3. Cell Aggressiveness

Increased aggressiveness of BC cells enhances the malignancy of tumors, including cell invasion, migration, and distant metastasis. The ability of BC cells to invade and migrate enables them to change their location within tissues and separate from the primary tumor, thus spreading. It also allows tumor cells to enter the circulation along the blood vessels and lymphatic vessels, and then reach distant body organs for colonization, that is, metastasis [72]. It is reported that EMT plays a key role in this process [73]. The whole metastasis process is also regulated by a complex set of signaling pathways, some of which are abnormally activated by MSC-EVs. Studies have demonstrated that both ERK and Wnt signaling pathways can be activated by MSC-EVs, which then exert a positive effect on EMT and migration of BC cells [57,60]. The secretion of exosomes promoting metastasis is sometimes under the regulation of other factors. A study demonstrated that the type 2 diabetes mellitus (T2DM) can alter the phenotype of AT-MSC-exos, causing them to exhibit a greater induction of the upregulation of genes associated with BC cell migration (e.g., C-X-C receptor 4 (CXCR4) and vascular endothelial growth factor C (VEGF)-C) and genes associated with BC cell metastasis (e.g., transforming growth factor-beta (TGF-β), basic fibroblast growth factor (bFGF) and epidermal growth factor (EGF)) [74]. In addition, some special cargoes loaded by EVs can also facilitate BC cell aggressiveness. Transwell assays and wound-healing assays revealed that the invasion and migration capacity of TNBC cells were both accelerated by MSC-exosomal-miR-106a-5p, but this effect could be inhibited by lncRNA HAND2-AS1 [63].

On the contrary, the contents of MSC-EVs also own the ability to suppress the aggressiveness of BC cells. For instance, the tripartite motif 59 (TRIM59) gene, as a marker of tumorigenesis, is highly expressed in BC cells and is strongly associated with proliferation and migration. Simultaneously, it can be inhibited as a target of hUC-MSC-exosomal-miR-148b-3p to suppress the invasion and migration of MDA-MB-231 cells [65]. Similarly, hUC-MSC-exosomal-miR-21-5p directly inhibits zinc finger protein 367 (ZNF367) expression by binding on its 3′UTR, and the invasion and migration ability of MCF-7 promoted by ZNF367 were attenuated after internalizing the hUC-MSC-exos [75]. Furthermore, Shojaei et al. cocultured AT-MSCs-exos with MDA-MB-231 cells and they discovered that exosome-loaded miR-381-3p can downregulate the essential genes (LRP6 and CTNNB1) in the Wnt signaling pathway, which can promote BC cell proliferation and migration, as well as the EMT transcription factors (Twist and Snail) to exert an inhibitory effect on invasion and migration of TNBC cells [76]. Additionally, miR-let-7f levels in BMSCs and their exosomes were upregulated by stromal cell-derived factor-1alpha (SDF-1α) treatment, hypoxia, or autophagy induction. BMSC-exos with overexpressed miR-let-7f can be incorporated into BC cells to impair the proliferation and migration of 4T1 BC cells [77]. 

In sum, the migration and metastasis ability of BC cells are not only regulated by aberrant signaling pathways or diseases, such as tumor T2DM, but are also under the control of the types of cargoes in MSC-EVs.

### 2.4. Angiogenesis

Angiogenesis is the process by which new capillaries are generated from pre-existing blood vessels, which removes disposals and provides nutrients for cancer cells, hence playing an essential role in the development of BC [78,79]. A wide range of factors in BC have been demonstrated to destroy the angiogenic balance, thereby switching on the angiogenic process and promoting cancer progression [80,81]. MSC-EVs containing angiogenic stimulatory factors, such as VEGF and FGF, can promote angiogenesis [82]. As a key mediator of angiogenesis in cancer, VEGF is upregulated by oncogene expression, a variety of growth factors, and hypoxia, and can promote mitosis, anti-apoptosis, cell migration, and increase vascular permeability [83,84]. It has been proven that MCF-7 cells co-injected with BMSC-EVs have higher angiogenesis ability [61]. However, based on the analysis of existing studies, MSC-EVs regulate BC angiogenesis mainly through the targeting effects of some miRNAs on VEGF in BC. Lee et al. have demonstrated that BMSC-exos could be internalized by BC cells and significantly downregulate the expression of VEGF in BC, thereby inhibiting angiogenesis. Furthermore, this effect of VEGF downregulation was due to the enrichment of miR-16 (a miRNA known to target VEGF) in MSC-exos [85]. What is more, VEGF is also regulated by the transcription factor hypoxia-inducible factor-1alpha (HIF-1α) through binding to the hypoxia response element within the VEGF gene promoter [86]. Rapamycin (mTOR), a key regulator of endothelial cell proliferation and angiogenesis [87], plays a central role in the HIF-1α-mediated expression of VEGF in BC cells [88]. Further research has demonstrated that BMSC-exos can also transfer miR-100 to decrease the expression and secretion of VEGF through modulating the mTOR/HIF-1α axis, thereby inhibiting angiogenesis in BC cells. Moreover, MSCs-derived exosomal miR-100 downregulated VEGF in a dose-dependent and time-dependent manner [89]. All in all, most of the existing evidence indicates that MSC-EVs have an inhibitory effect on BC angiogenesis, and only a few studies suggest the promoting effect.

### 2.5. Immune Regulation

Immune escape or immunosuppression is a vital part of the development of BC. Mechanisms by which cancer cells evade immune surveillance may include decreasing immunogenicity and immunosuppressive signals [90,91]. Immune checkpoint, a mechanism that regulates the normal activation of the immune system, inhibit the function of effector T cells upon encountering cancer cells containing ligands, such as programmed cell death ligand-1 (PD-L1) and cytotoxic T-lymphocyte-associated protein 4 (CTLA-4), thereby promoting the escape of cancer cells from immune surveillance [92]. In recent years, the successful application of immune checkpoint therapy in various cancers has aroused further interest in the study of cancer immune dysregulation, including the modification of tumor immunity by EVs. EVs play an important role in immune regulation between immune and non-immune cells through meditators expressed on their surface or transported in the lumen [93]. In BC, a study has demonstrated that human and mouse tumor-educated MSC-exos can accelerate the malignant growth by inducing the differentiation of monocytic myeloid-derived suppressor cells (M-MDSC) into highly immunosuppressive M2-polarized macrophages at tumor beds, and both M-MDSC cells and M2-polarized macrophages can promote the invasive ability and EMT of BC cells. Mechanistically, TGF-β, C1q, and semaphorins contained in the MSC-exos can promote myeloid tolerance not only by driving the overexpression of PD-L1 in immature myelomonocytic precursors and committed CD206+ macrophage but also by inducing differentiation of MHC class II+ macrophages with enhanced L-Arginase activity and IL-10 secretion at tumor beds. Both abilities can augment the progression of BC [94]. On the contrary, there is also evidence that MSC-EVs are involved in inducing immune responses. For instance, it was reported that AT-MSC-EVs with overexpressed human IL-2, a cytokine that regulates immune cells activation and proliferation, can stimulate the proliferation of CD8+ T-killer cells, thereby effectively killing TNBC cells [95]. In conclusion, MSC-EVs may act as both stimulatory and/or inhibitory mediators of immune cells in BC.

### 2.6. Dormancy

As a part of tumor progression, dormancy is of great significance for tumor metastasis and recurrence, including two central concepts: cellular dormancy and tumor mass dormancy [96]. Cellular dormancy is the ability of an individual cancer cell to enter a state of temporary cell-cycle arrest, characterized by three features: minimum proliferation, minimum death, and reversibility [97,98]. Whereas tumor mass dormancy is a condition in which the proliferation and death of tumor cells are in balance and the whole tumor mass does not expand, this process is associated with cellular hypoxia, limited vascularization, and/or removal of proliferating cells by immune cells, hence it is also termed “angiogenic dormancy” and “immune dormancy” [96,99,100]. It is reported that disseminated BC cells have the potential to metastasize to the bone marrow (BM) and activate a dormant state in a microenvironment rich in MSC-EVs. Treatment of MCF7 cells with MSC-EVs initiated an epithelial cell phenotype with reduced migration, decreased cell proliferation, and enhanced adhesion, which collectively supported BC dormancy [101]. As a niche, the TME plays an important role in inducing cancer cells into dormancy. One of the main concerns on the role of MSC-EVs is that MSCs could migrate to TME and interact with BC cells by secreting exosomes containing BC dormancy promoting miRNAs. For example, Mohd Ali et al. co-cultured MCF7-luminal and MDA-basal cells subtypes with AT-MSCs, then isolated MSC-exos and found that the interaction of MSCs with cancer cells resulted in different exosomal miRNAs profiles. Further studies have demonstrated that AT-MSC-derived exosomal miR-941, as the dormancy signatures for MCF7 and MDA cells, increased E-cadherin and decreased vimentin, SMAD4, and SNAIL expression to inhibit EMT and metastasis, which arrest the cell cycle into dormancy [102]. In another study, co-culturing of BM-metastatic human BC cell line (BM2) with BMSCs isolated from human donors revealed an increase of various miRNAs in BMSC-exos compared with those from adult fibroblasts and transferred to BM2 cells. Overexpression of miR-23b induced dormant phenotypes via inhibiting the target gene, myristoylated alanine-rich C-kinase substrate (MARCKS), which encodes a protein that promotes cell cycling and motility [103]. Moreover, dormant BC cells also have a function to induce more cancer cells to go dormant by inducing MSC to release miRNA-containing exosomes. miR-222 and miR-223 were upregulated in exosomes isolated from dormant BC-induced BMSCs and in turn, decreased CDK4, Cyclin D1 and p21WAF1 expression to initiate cycling quiescence of cancer cells and produce resistance to carboplatin, whereas the antagomiR-222/223 could reverse the quiescent phenotype of BC [104]. 

Another attractive phenomenon and mechanism by which MSC-EVs initiate BC cell dormancy is the MSC-EVs direct stepwise dedifferentiation of BC cells into dormancy, as cancer stem cells (CSCs). When BC cells entered the BM perivascular region and were contacted with MSCs, the contents of MSC-EVs completely dedifferentiated and changed into a population with similar properties as CSCs through the Wnt/β-catenin pathway. Additionally, BC cell progenitors are more sensitive to MSC-EVs, can also dedifferentiate in vivo, and express multipotent pathways similar to CSCs [105]. All the findings above indicated that MSC-EVs play roles in promoting BC dormancy.

### 2.7. Drug Resistance

**Figure 2:**
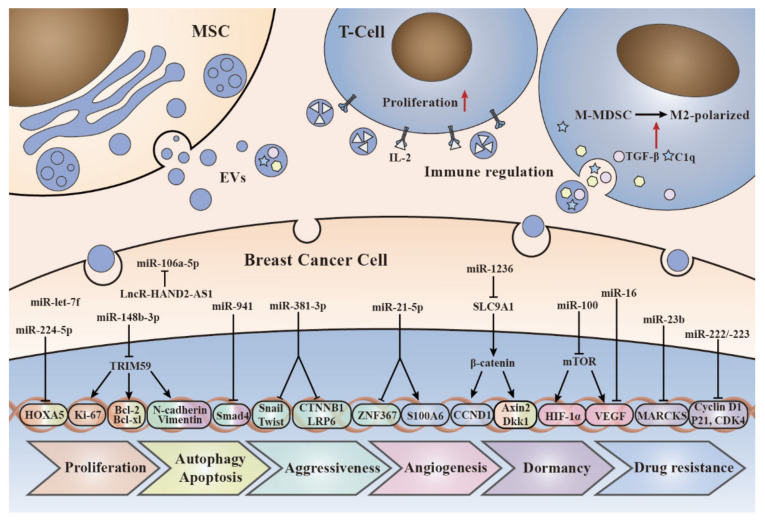
The molecular mechanisms of MSC-EVs cargos in breast cancer development. This figure depicts how the EVs interact with the recipient cells and the molecular mechanisms by which cargos loaded in EVs affect breast cancer cells, including proliferation, autophagy, apoptosis, aggressiveness, angiogenesis, immune regulation, dormancy, and drug resistance.

Drug resistance, defined as a decrease in the efficacy and potency of a drug to produce therapeutic merit, is a principal limiting factor in achieving cures for cancer and is considered to be one of the most critical factors affecting the prognosis of cancer patients [106]. Thirty percent of early BC patients develop metastatic disease, most of whom develop resistance to current chemotherapies [107]. When the tumor develops resistance, drugs lose their original efficacy and the disease recurs [108]. Despite advances in chemotherapy as the primary treatment for cancers [109], the roles of MSC-EVs in BC resistance remains unclear. A study suggests that MSC-exos contribute to chemoresistance in BC. Luo et al. have demonstrated that MSC-exos induced tolerance of BC cells to doxorubicin (Dox), while Dox-treated MSC-exos lead to greater resistance. Mechanistically, Dox induced the expression of miR-21-5p in MSCs and MSC-exos, and upregulated the expression of S100A6 in BC cells, thereby improving cell viability and Dox resistance [110]. However, Jia et al. drew an opposite conclusion that AT-MSC-exos significantly reduced the resistance of BC cells to cisplatin (DDP). Specifically, MSC-exos carrying miR-1236 can downregulate SLC9A1 expression and inactivate Wnt/β-catenin, ultimately improving the sensitivity of BC cells to DDP [111]. In sum, these data suggest that MSC-exos play a paradoxical role in mediating drug resistance in BC. Further research is needed to exploit the role of MSC-EVs in BC drug resistance. Altogether, we summarized the effects of MSC-EVs on cell proliferation, autophagy, apoptosis, aggressiveness, angiogenesis, immune regulation, dormancy and drug resistance of BC (Table 1 and Figure 2). 

## 3. The Potential Therapeutic Strategies of Mesenchymal Stem Cell-Derived Extracellular Vesicles in Breast Cancer

Current treatments of BC include surgical treatment, chemotherapy, radiotherapy, hormone therapy, and target therapy [112]. Surgical treatment is effective for early BC without metastasis but is prone to recur after surgery [103,113]. Chemotherapy and radiotherapy are adjuvant therapy to surgery, which often face drug resistance and several toxic side effects due to the inability to discriminate between rapidly dividing normal cells and cancer cells [114]. Hormone therapy is only suitable for patients with positive ERs and PRs, but not for receptor-negative cells, such as TNBC cells [115]. Obviously, all current treatments face challenges. Hence, to minimize or eliminate recurrence, drug resistance, and toxic effects, while ensuring that BC patients have a good quality of life, more attention should be paid to the research of novel therapies for BC. In what follows, we will discuss the role of MSC-EVs in the treatment of BC.

### 3.1. Mesenchymal Stem Cell-Derived Extracellular Vesicles as Drug Carriers for Breast Cancer Therapy

EVs have biophysical properties, such as biocompatibility, low toxicity, low immunogenicity, enhanced circulation stability, and bio-barrier permeability. Therefore, they can be used as effective drug carriers to improve the regulation of target tissues and organs [116,117]. In addition, MSCs are a kind of stem cells derived from the patients themselves and have the advantages of being non-invasive, non-toxic, easy to isolate, and with a high capacity for in vitro expansion; besides, they can produce a large number of stable exosomes with regenerative properties, which has facilitated the use of MSCs in this field [70,118,119]. It is reported that the continuously proliferating human MSC544 cell line is superior to the growth-restricted primary MSCs, providing an unlimited source for large-scale exosome production [120]. MSC-EVs can deliver different types of cargoes, such as natural products and chemotherapeutic agents and can target specific cells for the treatment of BC. 

**Figure 3:**
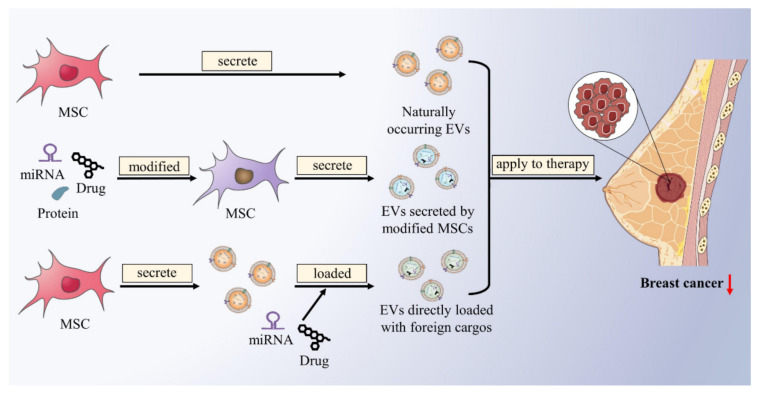
Naturally occurring and engineered MSC-EVs loaded with endogenous or exogenous cargos for therapeutic purposes. Naturally occurring MSC-EVs contain functional components that inhibit BC development. MSCs can be modified to produce EVs loaded with miRNAs, proteins, or anticancer drugs. Exogenous cargos, such as miRNA and chemotherapeutic drugs, can be loaded into MSC-EVs through engineering techniques. Naturally occurring and engineered MSC-EVs can act on BC to produce therapeutic effects.

Therapeutic MSC-EVs include naturally occurring MSC-EVs, EVs secreted by modified MSCs, and MSC-EVs directly loaded with foreign cargos (Figure 3). Firstly, for naturally occurring MSC-EVs, it has been proven above that some endogenously produced MSC-EVs contain functional components that inhibit BC development. For example, AT-MSC-exos transferred miR-145 to BC cells and can increase miR-145 levels in BC cells, which can induce apoptosis and inhibit metastasis of BC by modulating the Rho-Associated Coiled-Coil Containing Protein Kinase 1 (ROCK1), Erb-B2 Receptor Tyrosine Kinase 2 (ERBB2), Matrix Metalloproteinase 9 (MMP9), and Tumor Protein p53 (TP53) expression [121]. Using MSC-EVs that contain endogenous BC suppressors may be a potential new therapeutic strategy for BC. Secondly, the content of MSC-EVs can vary in response to different physiological and pathological conditions [122,123,124]. MSCs can be modified by pathological factors, transfection, and drug loading to produce EVs loaded with miRNAs, proteins, or anticancer drugs to treat BC. Vakhshiteh et al. transfected DPSC with the BC inhibitor miR-34a, then used DPSC-exos as a good carrier for delivery to MDA-MB-231 cells, and then observed enhanced apoptosis and reduced invasion and migration [125]. Alternatively, MSCs have an ability for tumor homing, which has been proven to evade the immune response and home to the site of metastasis. miR-379 acts as a potent BC suppressor, can significantly reduce lymph node metastasis partly mediated by modulating the cyclooxygenase-2 (COX-2). O’Brien and colleagues found that only miR-379 derived from MSC-EVs showed a reduction in tumor size rather than MSC transfected with miR-379. Altogether, engineered tumor-homing MSC cells to secrete miR-379-enriched EVs as tumor-targeted delivery vehicles were demonstrated to be an innovative therapy for the metastatic BC [126]. In addition to miRNAs, a chemotherapeutic drug for BC, the paclitaxel (Taxol), has also been studied similarly. Kalimuthu et al. mixed MSCs with Taxol at different concentrations in phosphate-buffered saline (PBS) and used simple procedures to isolate Taxol-loaded MSC-exo mimetics, which demonstrated therapeutic efficiency for the treatment of BC both in vitro and in vivo [127]. Another study demonstrated that Taxol transported via MSC-exos achieved the same cytotoxic effects and inhibition of metastasis at a reduced concentration compared to direct use. These findings indicate that drug-loaded MSC-exos have a specific and more efficient tumor-targeting property [128]. Thirdly, through EVs engineering techniques, such as electroporation, lipofection, sonication or extrusion, exogenous cargos, such as miRNA and chemotherapeutic drugs, can be loaded into MSC-EVs to exert antitumor effects [129]. For instance, locked nucleic acid (LNA)-modified anti-miRNA oligonucleotides can be incorporated into MSC-EVs to inhibit tumor-promoting miRNAs and thus suppress BC [130]. Naseri et al. encapsulated LNA-antimiR-142-3p into MSC-exos for efficient delivery to MCF7-derived cancer stem-like cells, thereby reducing the expression levels of miR-142-3p and miR-150 to targeted increase expression of the genes APC and P2X7R, further leading to reduced clone formation and tumor initiation of MCF7-derived cancer stem-like cells [131]. It was reported that CD90low AT-MSCs-EVs exert antitumor effect in tumor-bearing mice BC compared with CD90high AT-MSCs; moreover, the antitumor activity of CD90low AT-MSC-EVs loaded with the antioncogenic miRNA-16-5p was significantly enhanced, suggesting that AT-MSC-EVs can not only be applied as an antitumor model to preclinical treatment of breast cancer, but also as a carrier to transfer cargos and enhance their function [132]. Dox is a widely used BC chemotherapeutic drug; however, a large number of side effects, such as congestive heart failure and drug resistance, exist among patients taking Dox [133]. Gomari et al. have found that Dox can be packed in MSC-exos for targeted delivery to TUBO cancer cells (HER-2-positive murine BC cells); this targeted process is accomplished by the binding of LAMP2b-DARPin protein on the surface of exosomes to HER2 protein. The results demonstrated that cytotoxicity of targeted Dox-loaded exosomes was higher than that of free Dox at 72 h and that the specific delivery of Dox in the target tissues of the murine BC model was via targeted exosomes versus untargeted exosomes. This study revealed that targeted delivery of MSC-exos loaded with Dox markedly improved drug efficacy and reduced side effects, such as myelosuppression and cardiotoxicity [119] (Table 2).

As small natural membrane-based delivery vectors, MSC-EVs can not only act as a carrier for BC in situ, but also cross the blood–brain barrier, which offers a new idea for the treatment of BC brain metastasis. CXCR4 is a chemokine receptor used to mediate the targeting of MSCs to tumor cells, whereas tumor necrosis factor-related apoptosis-inducing ligand (TRAIL) is used to mediate the induction of apoptosis, as it can selectively induce apoptosis of transformed cells without obvious toxic side effects in normal tissues. CXCR4/TRAIL-enriched MSC-exos exerted pronounced activity against brain metastasis from BC as a cooperator of carboplatin in vivo. Together, ExoCXCR4+TRAIL not only has potential as a therapeutic tool for BC brain metastasis but also highlights a new prospect for establishing a synergistic regimen with anticancer drugs to treat brain disease [134].

### 3.2. Other Studies on the Application of Mesenchymal Stem Cell-Derived Extracellular Vesicles in the Treatment of Breast Cancer

It is known that conventional BC chemotherapy has several drawbacks, the most serious of which is caused by the difficulty of localizing the drug to kill tumor cells. There is evidence that MVs derived from AT-MSCs can induce apoptosis in human BC cells. Based on previous experience, the optimal effect of the drug depends on its precise concentration and duration of activation. MSC-MVs can be packed into polycaprolactone (PCL) nanofibers and thus released for a long time, and this apoptotic effect is maintained in cancer cells cultured on PCL-MVs nanofibers, suggesting that AT-MSC-MVs can be used as an in situ inhibitor rather than as a chemo-drug [70]. Similarly, Chulpanova et al. reported that cytochalasin B was also capable of inducing MSCs to release EVs for increasing the yield of EVs [95]. In addition, BC patients after lumpectomy may require augmentation or surgical cosmetic procedure with scaffolds containing MVs, which have two potential advantages: the ability to fill the cavity created by the removal of cancerous breast tissue as well as causing the death of probable BC cells [70].

Although current research into the application of MSC-EVs is still in the preclinical stage, MSC-EVs provide solutions to many challenges faced by clinicians in the treatment of BC, which is expected to become a new hope for BC therapy.

## 4. Discussion and Conclusions

As kinds of stromal cells, MSCs have been reviewed for their crucial role in cancer progression, and their important applications in cancer therapy have also been proposed [33,135]. With in-depth research, EVs, as a research hotspot in recent years, are emerging as major players in the communication between MSCs and tumor cells. Both Vakhshiteh et al. and Zhou et al. reviewed the promotive and inhibitory effects of MSC-exos at different stages of cancer progression and their application as drug delivery vehicles in cancer therapy [136,137]. The related review of Weng and colleagues focused more on the specific operation of MSC-EVs in the application of cancer treatment [138]. Although many reviews on the role of MSC-EVs in cancer progression and treatment have been published, no one has ever focused on one specific cancer, BC, which is one of the most common malignancies in women, but the current treatment methods adopted are still relatively conventional techniques with only passable results. In order to better eradicate BC, a novel efficient and precise treatment strategy is needed. Based on extensive previous research, we found the special role of MSC-EVs in the development of BC. We think it is of great necessity to review the relationship between MSC-EVs and BC, as this may provide a new perspective on BC treatment. For the first time, in light of the characteristics of breast cancer, we systematically reviewed the dual roles of MSC-EVs in BC growth, metastasis, angiogenesis, immune regulation, dormancy, and drug resistance, as well as their potential therapeutic strategies in BC, thereby enabling us to treat BC more effectively.

The above descriptions reveal an interesting conclusion that MSC-EVs have dual roles in the development of BC. Previous evidence has demonstrated that MSCs can promote and suppress BC progression, and the dual roles of MSC-EVs are similar to MSCs themselves [139,140]. As all the conclusions come from experimental results, the contradictory findings may be due to the variances in different experimental operations. For example, differences in the timing of MSC growth, the composition of culture medium, and the passage of MSC, as well as unclear exosome quality control during harvesting/sample preparation, may influence the results [140]. In addition, the paracrine function of MSCs can be regulated by oxygen tension; thus, exosome cargo differences may exist in MSCs grown under variable oxygen tension [141]. Likewise, the growth time for MSC-exos isolation and the quality of exosome depleted serum in MSC growth can both affect the experimental results [140]. More notably, MSCs from different sources may secrete EVs consisting of different cargoes, thus affecting their effect on BC cells. For instance, AT-MSC-exos can promote BC development, while hUC-MSC-exos can inhibit BC progression [60,65]. Furthermore, AT-MSC-exos promoted the migration of luminal BC (MCF7 cells) but suppressed the aggression of negative BC (MDA-MB-231 cells), suggesting that MSC-exos may have opposite effects on different types of BC [60,76]. Altogether, we can speculate that MSC-EVs are multifaceted regulators of BC progression and are influenced by multiple factors.

Although we have separately summarized the role of MSC-EVs on each stage of tumor progression in this review, the effects of MSC-EVs on BC development are continuous and multi-process [142]. Cell apoptosis and cell proliferation are mutually restricted, whereas autophagy can promote cell renewal and inhibit cell apoptosis. The dormant state has a reduced capacity for both proliferation and migration, while invasion and migration underlie distant metastasis [143]. It follows that the tumorigenesis of BC is interconnected, and the influence of MSC-EVs is also multi-stage and continuous.

In addition to the growth of BC in situ, the roles of pre-metastatic niche (PMN) in BC development have also received wide attention in recent years. According to the well-known “Seed and Soil” theory proposed by Paget, tumor cells (seeds) can only grow in certain specific and permissive microenvironments (soil) [144]. PMN is such a favorable environment for metastatic tumor cells to colonize the second site, containing nutrients, remodeled extracellular matrix, and supporting signals from stromal cells [145,146]. MSCs are a subset of stromal cells that play an important role in PMN. Recently, however, it has also been proposed that exosomes can act as “fertilizers” and are essential to the establishment and maintenance of PMN [147]. MSC-exos contain a variety of molecules and factors that support tumor growth in vivo, participate in tumor cells acquiring anti-apoptosis and migration ability, and stimulate angiogenesis, thus promote the formation of PMN [148,149]. In Sanmartin’s review, it is systematically reported that MSCs and their EVs play a significant role in BM/bone PMN establishment for BC cell colonization [150]. However, there are rare studies on MSC-EVs roles in the formation of PMN in other organs of BC. Therefore, we hope to have more studies on the role of MSC-EVs in the formation of PMN in other organs of BC in the future, which will bring a major breakthrough for the treatment of distant metastasis of BC. 

Collectively, we have discussed the multiple effects of MSC-EVs on BC development and their therapeutic potential. However, research on MSC-EVs has been in the pre-clinical stage. Basic research and emerging technologies need to be integrated to fully exploit the potential of MSC-EVs. Despite some promising advances in using MSC-EVs as drug carriers, there is still a necessity to develop appropriate strategies and techniques to tailor MSC-EVs with high drug delivery capabilities, enhanced target specificity, and non-cytotoxic effects. We believe that summarizing the role of MSC-EVs in BC progression can serve as a basis for further clinical studies and provide new ideas for the diagnosis and treatment of BC. 

## Figures and Tables

**Table 1:** The role of MSC-EVs in breast cancer tumorigenesis.

Donor Cells	Cargo	Molecular Mechanism	Biological Functions	Reference
AT-MSCs	Apoptosis-induced factors and substances	Upregulate Bax, P53, E2F2 and SMAD5 genes, and downregulate bcl gene	Proliferation↓;apoptosis↑	[70]
AT-MSCs	miR-381-3p	Downregulate Wnt signaling pathway genes (LRP6 and CTNNB1) and EMT transcription factors (Twist and Snail)	Invasion, migration↓	[76]
AT-MSCs	IL-2	Stimulate proliferation of CD8+ T-killer cells	Immunity↑	[95]
AT-MSCs	miR-941	Increased E-cadherin and decreased vimentin, SMAD4 and SNAI1 expression to inhibit EMT and metastasis	Dormancy↑;metastasis↓	[102]
AT-MSCs	miR-1236	Downregulate SLC9A1 expression to inactivate Wnt/β-catenin signaling pathway and downregulate CCND1 expression	Drug resistance↓	[111]
AT-MSCs	ND	Activate Wnt signaling pathway, increase β-catenin expression, and upregulate Wnt target genes, such as Axin2 and Dkk1	Proliferation, migration↑	[60]
AT-MSCs	ND	Upregulate CXCR4, VEGF-C, TGF-β, bFGF and EGF	Migration, metastasis↑	[74]
BMSCs	miR-106a-5p	ND	Proliferation↑	[63]
BMSCs	miR-let-7f	ND	Proliferation, migration↓	[77]
BMSCs	miR-16	Downregulate VEGF expression	Angiogenesis↓	[85]
BMSCs	miR-100	Inhibit mTOR/HIF-1α axis to downregulate VEGF expression	Angiogenesis↓	[89]
BMSCs	miR-23b	Inhibit MARCKS gene	Dormancy↑	[103]
BMSCs	miR-222, miR-223	Decrease CDK4, Cyclin D1 and p21WAF1 expression to initiate BC cells cycling quiescence	Dormancy↑	[104]
hUC-MSCs	miR-224-5p	Inhibit HOXA5 gene	Proliferation, autophagy↑;apoptosis↓	[62]
hUC-MSCs	miR-148b-3p	Inhibit TRIM59 gene to downregulate Ki-67, Bcl-xl, Bcl-2, N-cadherin and vimentin expression	Proliferation, invasion, migration↓;apoptosis↑	[65]
hUC-MSCs	miR-21-5p	Downregulate ZNF367 expression	Invasion, migration↓	[75]
hUC-MSCs	ND	Activate ERK signaling pathway to induce EMT, increase N-cadherin expression and decrease E-cadherin expression	Proliferation, migration↑	[57]
MSCs	TGF-β, C1q, semaphroins	Upregulate PD-L1 expression and increase L-Arginase and IL-10 to induce M-MDSC differentiation into M2-polarized macrophages for immunosuppressive	Immunity↓;invasion↑	[94]
MSCs	miR-21-5p	Upregulate S100A6 expression	Drug resistance↑	[110]
MSCs	ND	Regulate Wnt/β-catenin pathway to dedifferentiate BC cells into CSCs	Dormancy↑	[105]

ND, not determined; ↓, suppressing effect; ↑, promoting effect.

**Table 2:** The clinical role of MSC-EVs in breast cancer.

Cargos	Molecular Mechanisms	Biological Functions	Classification	Reference
miR-145	Modulating ROCK1, MMP9, ERBB2, and TP53 expression	apoptosis↑; metastasis↓	Naturally occurring EVs	[121]
miR-34a	ND	apoptosis↑; invasion, migration↓	EVs secreted by modified MSCs	[125]
miR-379	Modulating COX-2	metastasis↓	EVs secreted by modified MSCs	[126]
Taxol	ND	Cytotoxicity↑ (in vitro); tumor growth↓ (in vivo)	EVs secreted by modified MSCs	[127]
LNA-antimiR-142-3p	Reducing miR-142-3p and miR-150 to targeted increase expression of genes APC and P2X7R	Apoptosis, proliferation↑; cancer stem-like cells clonogenicity and tumorigenicity↓	EVs directly loaded with foreign cargos	[131]
miRNA-16-5p	ND	apoptosis↑ (in vitro); tumor growth↓ (in vivo)	EVs directly loaded with foreign cargos	[132]
Dox	Binding to HER2 protein	Drug efficacy↑; side effects (myelosuppression and cardiotoxicity) ↓	EVs directly loaded with foreign cargos	[119]

ND, not determined; ↑, promoting effect; ↓, suppressing effect.

## Abbreviations

AT-MSC-exos: adipose tissue-derived mesenchymal stem cells-derived exosomes; AT-MSCs: adipose tissue-derived mesenchymal stem cells; BC: breast cancer; bFGF: basic fibroblast growth factor; BM: bone marrow; BM2: bone marrow-metastatic human BC cell line; BMSCs: bone marrow-derived mesenchymal stem cells; COX-2: Cyclooxygenase-2; CSCs: cancer stem cells; CTLA-4: cytotoxic T-lymphocyte-associated protein 4; CXCR4: C-X-C receptor 4; DDP: cisplatin; DNMT3a: DNA methyltransferase 3a; Dox: doxorubicin; DPSC: dental pulp-derived mesenchymal stem cells; EEs: early endosomes; EGF: epidermal growth factor; EMT: epithelial-mesenchymal transition; ER: estrogen receptor; ERBB2: Erb-B2 Receptor Tyrosine Kinase 2; ERK: extracellular signal regulated kinase; ESCs: embryonic stem cells; EVs: extracellular vesicles; FAK: focal adhesion kinase; HDGF: hepatoma-derived growth factor; HER-2: human epidermal growth factor receptor-2; HIF-1α: hypoxia-inducible factor-1 alpha; hMSCs: human mesenchymal stem cells; hWJMSCs: human Wharton’s jelly mesenchymal stem cells; IL: interleukin; ILVs: intraluminal vesicles; LNA: locked nucleic acid; lncRNA HAND2-AS1: long non-coding RNA heart and neural crest derivatives expressed 2-antisense RNA 1; MAPK: mitogen-activated protein kinase; MARCKS: Myristoylated Alanine-Rich C-kinase Substrate; MCP-1: monocyte chemotactic protein-1; miRNA: microRNA; MMP9: Matrix Metalloproteinase 9; M-MDSCs: monocytic myeloid-derived suppressor cells; mRNA: messenger RNA; MSC-EVs: mesenchymal stem cell derived extracellular vesicles; MSC-exos: mesenchymal stem cell-derived exosomes; MSC-MVs: mesenchymal stem cell derived microvesicles; MSCs: mesenchymal stem cells; mTOR: Rapamycin; MVBs: multivesicular bodies; MVs: microvesicles; PBS: phosphate-buffered saline; PCL: Polycaprolactone; PD-L1: programmed cell death ligand-1; PMN: pre-metastatic niche; PR: progesterone receptor; PRC1: polycomb repressor complex 1; PTEN: phosphatase and tensin homolog deleted on chromosome 10; ROCK1: Rho-Associated Coiled-Coil Containing Protein Kinase 1; SDF-1α: stromal cell-derived factor-1 alpha; siRNA: small interfering RNA; T2DM: type 2 diabetes mellitus; Taxol: paclitaxel; TGF-β: transforming growth factor-beta; TME: tumor microenvironment; TNBC: triple negative breast cancer; TP53: Tumor protein p53; TRAIL: tumor necrosis factor-related apoptosis-inducing ligand; TRIM59: tripartite motif 59; UC-MSCs: umbilical cord-derived mesenchymal stem cells; uPA: urokinase plasminogen activator; VEGF: vascular endothelial growth factor; ZNF367: zinc finger protein 367.

## References

[1] Azamjah N., Soltan-Zadeh Y., Zayeri F. (2019). Global Trend of Breast Cancer Mortality Rate: A 25-Year Study. Asian Pac. J. Cancer Prev. APJCP.

[2] Wilkinson L., Gathani T. (2022). Understanding breast cancer as a global health concern. Br. J. Radiol..

[3] Sung H., Ferlay J., Siegel R.L., Laversanne M., Soerjomataram I., Jemal A., Bray F. (2021). Global Cancer Statistics 2020: GLOBOCAN Estimates of Incidence and Mortality Worldwide for 36 Cancers in 185 Countries. CA A Cancer J. Clin..

[4] Tsang J.Y.S., Tse G.M. (2020). Molecular Classification of Breast Cancer. Adv. Anat. Pathol..

[5] Thakur V., Kutty R.V. (2019). Recent advances in nanotheranostics for triple negative breast cancer treatment. J. Exp. Clin. Cancer Res. CR.

[6] Dees S., Ganesan R., Singh S., Grewal I.S. (2021). Bispecific Antibodies for Triple Negative Breast Cancer. Trends Cancer.

[7] Kwapisz D. (2021). Pembrolizumab and atezolizumab in triple-negative breast cancer. Cancer Immunol. Immunother. CII.

[8] Yin L., Duan J.J., Bian X.W., Yu S.C. (2020). Triple-negative breast cancer molecular subtyping and treatment progress. Breast Cancer Res. BCR.

[9] Trayes K.P., Cokenakes S.E.H. (2021). Breast Cancer Treatment. Am. Fam. Physician.

[10] Barzaman K., Karami J., Zarei Z., Hosseinzadeh A., Kazemi M.H., Moradi-Kalbolandi S., Safari E., Farahmand L. (2020). Breast cancer: Biology, biomarkers, and treatments. Int. Immunopharmacol..

[11] Abels E.R., Breakefield X.O. (2016). Introduction to Extracellular Vesicles: Biogenesis, RNA Cargo Selection, Content, Release, and Uptake. Cell. Mol. Neurobiol..

[12] Doyle L.M., Wang M.Z. (2019). Overview of Extracellular Vesicles, Their Origin, Composition, Purpose, and Methods for Exosome Isolation and Analysis. Cells.

[13] Bebelman M.P., Smit M.J., Pegtel D.M., Baglio S.R. (2018). Biogenesis and function of extracellular vesicles in cancer. Pharmacol. Ther..

[14] Zha Q.B., Yao Y.F., Ren Z.J., Li X.J., Tang J.H. (2017). Extracellular vesicles: An overview of biogenesis, function, and role in breast cancer. Tumour Biol..

[15] Borges F.T., Reis L.A., Schor N. (2013). Extracellular vesicles: Structure, function, and potential clinical uses in renal diseases. Braz. J. Med. Biol. Res..

[16] Kletukhina S., Neustroeva O., James V., Rizvanov A., Gomzikova M. (2019). Role of Mesenchymal Stem Cell-Derived Extracellular Vesicles in Epithelial-Mesenchymal Transition. Int. J. Mol. Sci..

[17] Park J.E., Tan H.S., Datta A., Lai R.C., Zhang H., Meng W., Lim S.K., Sze S.K. (2010). Hypoxic tumor cell modulates its microenvironment to enhance angiogenic and metastatic potential by secretion of proteins and exosomes. Mol. Cell. Proteom. MCP.

[18] Higginbotham J.N., Demory Beckler M., Gephart J.D., Franklin J.L., Bogatcheva G., Kremers G.J., Piston D.W., Ayers G.D., McConnell R.E., Tyska M.J. (2011). Amphiregulin exosomes increase cancer cell invasion. Curr. Biol. CB.

[19] Ciravolo V., Huber V., Ghedini G.C., Venturelli E., Bianchi F., Campiglio M., Morelli D., Villa A., Della Mina P., Menard S. (2012). Potential role of HER2-overexpressing exosomes in countering trastuzumab-based therapy. J. Cell. Physiol..

[20] Tourneur L., Mistou S., Schmitt A., Chiocchia G. (2008). Adenosine receptors control a new pathway of Fas-associated death domain protein expression regulation by secretion. J. Biol. Chem..

[21] Barile L., Vassalli G. (2017). Exosomes: Therapy delivery tools and biomarkers of diseases. Pharmacol. Ther..

[22] Kalluri R., LeBleu V.S. (2020). The biology, function, and biomedical applications of exosomes. Science.

[23] Atiya H., Frisbie L., Pressimone C., Coffman L. (2020). Mesenchymal Stem Cells in the Tumor Microenvironment. Adv. Exp. Med. Biol..

[24] Yekula A., Yekula A., Muralidharan K., Kang K., Carter B.S., Balaj L. (2019). Extracellular Vesicles in Glioblastoma Tumor Microenvironment. Front. Immunol..

[25] Maacha S., Bhat A.A., Jimenez L., Raza A., Haris M., Uddin S., Grivel J.C. (2019). Extracellular vesicles-mediated intercellular communication: Roles in the tumor microenvironment and anti-cancer drug resistance. Mol. Cancer.

[26] Goldstein R.H., Reagan M.R., Anderson K., Kaplan D.L., Rosenblatt M. (2010). Human bone marrow-derived MSCs can home to orthotopic breast cancer tumors and promote bone metastasis. Cancer Res..

[27] Komarova S., Roth J., Alvarez R., Curiel D.T., Pereboeva L. (2010). Targeting of mesenchymal stem cells to ovarian tumors via an artificial receptor. J. Ovarian Res..

[28] Hung S.C., Deng W.P., Yang W.K., Liu R.S., Lee C.C., Su T.C., Lin R.J., Yang D.M., Chang C.W., Chen W.H. (2005). Mesenchymal stem cell targeting of microscopic tumors and tumor stroma development monitored by noninvasive in vivo positron emission tomography imaging. Clin. Cancer Res..

[29] Shojaei S., Hashemi S.M., Ghanbarian H., Salehi M., Mohammadi-Yeganeh S. (2019). Effect of mesenchymal stem cells-derived exosomes on tumor microenvironment: Tumor progression versus tumor suppression. J. Cell. Physiol..

[30] Gubert F., da Silva J.S., Vasques J.F., de Jesus Gonçalves R.G., Martins R.S., de Sá M.P.L., Mendez-Otero R., Zapata-Sudo G. (2021). Mesenchymal Stem Cells Therapies on Fibrotic Heart Diseases. Int. J. Mol. Sci..

[31] Ji J.F., He B.P., Dheen S.T., Tay S.S. (2004). Interactions of chemokines and chemokine receptors mediate the migration of mesenchymal stem cells to the impaired site in the brain after hypoglossal nerve injury. Stem Cells (Dayt. Ohio).

[32] Whiteside T.L. (2018). Exosome and mesenchymal stem cell cross-talk in the tumor microenvironment. Semin. Immunol..

[33] Ridge S.M., Sullivan F.J., Glynn S.A. (2017). Mesenchymal stem cells: Key players in cancer progression. Mol. Cancer.

[34] Karnoub A.E., Dash A.B., Vo A.P., Sullivan A., Brooks M.W., Bell G.W., Richardson A.L., Polyak K., Tubo R., Weinberg R.A. (2007). Mesenchymal stem cells within tumour stroma promote breast cancer metastasis. Nature.

[35] El-Haibi C.P., Karnoub A.E. (2010). Mesenchymal stem cells in the pathogenesis and therapy of breast cancer. J. Mammary Gland. Biol. Neoplasia.

[36] Ljujic B., Milovanovic M., Volarevic V., Murray B., Bugarski D., Przyborski S., Arsenijevic N., Lukic M.L., Stojkovic M. (2013). Human mesenchymal stem cells creating an immunosuppressive environment and promote breast cancer in mice. Sci. Rep..

[37] Zhang B., Yin Y., Lai R.C., Tan S.S., Choo A.B., Lim S.K. (2014). Mesenchymal stem cells secrete immunologically active exosomes. Stem Cells Dev..

[38] Dostert G., Mesure B., Menu P., Velot É. (2017). How Do Mesenchymal Stem Cells Influence or Are Influenced by Microenvironment through Extracellular Vesicles Communication?. Front. Cell Dev. Biol..

[39] Green R.M. (2007). Can we develop ethically universal embryonic stem-cell lines?. Nat. Rev. Genet..

[40] Ben-David U., Benvenisty N. (2011). The tumorigenicity of human embryonic and induced pluripotent stem cells. Nat. Rev. Cancer.

[41] Gutierrez-Aranda I., Ramos-Mejia V., Bueno C., Munoz-Lopez M., Real P.J., Mácia A., Sanchez L., Ligero G., Garcia-Parez J.L., Menendez P. (2010). Human induced pluripotent stem cells develop teratoma more efficiently and faster than human embryonic stem cells regardless the site of injection. Stem Cells (Dayt. Ohio).

[42] Jabbari N., Akbariazar E., Feqhhi M., Rahbarghazi R., Rezaie J. (2020). Breast cancer-derived exosomes: Tumor progression and therapeutic agents. J. Cell Physiol..

[43] Zhang X., Tu H., Yang Y., Fang L., Wu Q., Li J. (2017). Mesenchymal Stem Cell-Derived Extracellular Vesicles: Roles in Tumor Growth, Progression, and Drug Resistance. Stem Cells Int..

[44] Jiang S., Chen H., He K., Wang J. (2022). Human bone marrow mesenchymal stem cells-derived exosomes attenuated prostate cancer progression via the miR-99b-5p/IGF1R axis. Bioengineered.

[45] Jiang S., Mo C., Guo S., Zhuang J., Huang B., Mao X. (2019). Human bone marrow mesenchymal stem cells-derived microRNA-205-containing exosomes impede the progression of prostate cancer through suppression of RHPN2. J. Exp. Clin. Cancer Res. CR.

[46] Liu J., Feng Y., Zeng X., He M., Gong Y., Liu Y. (2021). Extracellular vesicles-encapsulated let-7i shed from bone mesenchymal stem cells suppress lung cancer via KDM3A/DCLK1/FXYD3 axis. J. Cell. Mol. Med..

[47] Zhao J., Lin H., Huang K. (2022). Mesenchymal Stem Cell-derived Extracellular Vesicles Transmitting MicroRNA-34a-5p Suppress Tumorigenesis of Colorectal Cancer Through c-MYC/DNMT3a/PTEN Axis. Mol. Neurobiol..

[48] Jia Y., Ding X., Zhou L., Zhang L., Yang X. (2021). Mesenchymal stem cells-derived exosomal microRNA-139-5p restrains tumorigenesis in bladder cancer by targeting PRC1. Oncogene.

[49] Ying H., Lin F., Ding R., Wang W., Hong W. (2020). Extracellular vesicles carrying miR-193a derived from mesenchymal stem cells impede cell proliferation, migration and invasion of colon cancer by downregulating FAK. Exp. Cell Res..

[50] Dong L., Pu Y., Zhang L., Qi Q., Xu L., Li W., Wei C., Wang X., Zhou S., Zhu J. (2018). Human umbilical cord mesenchymal stem cell-derived extracellular vesicles promote lung adenocarcinoma growth by transferring miR-410. Cell Death Dis..

[51] Chang L., Gao H., Wang L., Wang N., Zhang S., Zhou X., Yang H. (2021). Exosomes derived from miR-1228 overexpressing bone marrow-mesenchymal stem cells promote growth of gastric cancer cells. Aging.

[52] Choi D.W., Cho K.A., Kim J., Lee H.J., Kim Y.H., Park J.W., Woo S.Y. (2021). Extracellular vesicles from tonsil-derived mesenchymal stromal cells show anti-tumor effect via miR-199a-3p. Int. J. Mol. Med..

[53] Deng L., Wang C., He C., Chen L. (2021). Bone mesenchymal stem cells derived extracellular vesicles promote TRAIL-related apoptosis of hepatocellular carcinoma cells via the delivery of microRNA-20a-3p. Cancer Biomark. Sect. A Dis. Markers.

[54] Chen Z., Xie Y., Chen W., Li T., Chen X., Liu B. (2021). microRNA-6785-5p-loaded human umbilical cord mesenchymal stem cells-derived exosomes suppress angiogenesis and metastasis in gastric cancer via INHBA. Life Sci..

[55] Liu L., Yu T., Jin Y., Mai W., Zhou J., Zhao C. (2021). MicroRNA-15a Carried by Mesenchymal Stem Cell-Derived Extracellular Vesicles Inhibits the Immune Evasion of Colorectal Cancer Cells by Regulating the KDM4B/HOXC4/PD-L1 Axis. Front. Cell Dev. Biol..

[56] Li S., Yan G., Yue M., Wang L. (2021). Extracellular vesicles-derived microRNA-222 promotes immune escape via interacting with ATF3 to regulate AKT1 transcription in colorectal cancer. BMC Cancer.

[57] Zhou X., Li T., Chen Y., Zhang N., Wang P., Liang Y., Long M., Liu H., Mao J., Liu Q. (2019). Mesenchymal stem cell-derived extracellular vesicles promote the in vitro proliferation and migration of breast cancer cells through the activation of the ERK pathway. Int. J. Oncol..

[58] Platanias L.C. (2003). Map kinase signaling pathways and hematologic malignancies. Blood.

[59] Lustig B., Behrens J. (2003). The Wnt signaling pathway and its role in tumor development. J. Cancer Res. Clin. Oncol..

[60] Lin R., Wang S., Zhao R.C. (2013). Exosomes from human adipose-derived mesenchymal stem cells promote migration through Wnt signaling pathway in a breast cancer cell model. Mol. Cell. Biochem..

[61] Vallabhaneni K.C., Penfornis P., Dhule S., Guillonneau F., Adams K.V., Mo Y.Y., Xu R., Liu Y., Watabe K., Vemuri M.C. (2015). Extracellular vesicles from bone marrow mesenchymal stem/stromal cells transport tumor regulatory microRNA, proteins, and metabolites. Oncotarget.

[62] Wang Y., Wang P., Zhao L., Chen X., Lin Z., Zhang L., Li Z. (2021). miR-224-5p Carried by Human Umbilical Cord Mesenchymal Stem Cells-Derived Exosomes Regulates Autophagy in Breast Cancer Cells via HOXA5. Front. Cell Dev. Biol..

[63] Xing L., Tang X., Wu K., Huang X., Yi Y., Huan J. (2020). LncRNA HAND2-AS1 suppressed the growth of triple negative breast cancer via reducing secretion of MSCs derived exosomal miR-106a-5p. Aging.

[64] Mirabdollahi M., Sadeghi-Aliabadi H., Haghjooy Javanmard S. (2020). Human Wharton’s jelly mesenchymal stem cells-derived secretome could inhibit breast cancer growth in vitro and in vivo. Iran. J. Basic Med. Sci..

[65] Yuan L., Liu Y., Qu Y., Liu L., Li H. (2019). Exosomes Derived From MicroRNA-148b-3p-Overexpressing Human Umbilical Cord Mesenchymal Stem Cells Restrain Breast Cancer Progression. Front. Oncol..

[66] Mariño G., Niso-Santano M., Baehrecke E.H., Kroemer G. (2014). Self-consumption: The interplay of autophagy and apoptosis. Nat. Rev. Mol. Cell Biol..

[67] Liu G., Pei F., Yang F., Li L., Amin A.D., Liu S., Buchan J.R., Cho W.C. (2017). Role of Autophagy and Apoptosis in Non-Small-Cell Lung Cancer. Int. J. Mol. Sci..

[68] Su Z., Yang Z., Xu Y., Chen Y., Yu Q. (2015). Apoptosis, autophagy, necroptosis, and cancer metastasis. Mol. Cancer.

[69] Daskalaki I., Gkikas I., Tavernarakis N. (2018). Hypoxia and Selective Autophagy in Cancer Development and Therapy. Front. Cell Dev. Biol..

[70] Rezaie Z., Ardeshirylajimi A., Ashkezari M.D. (2018). Improved anticancer properties of stem cells derived exosomes by prolonged release from PCL nanofibrous structure. Gene.

[71] Mirabdollahi M., Haghjooyjavanmard S., Sadeghi-Aliabadi H. (2019). An anticancer effect of umbilical cord-derived mesenchymal stem cell secretome on the breast cancer cell line. Cell Tissue Bank..

[72] Polacheck W.J., Zervantonakis I.K., Kamm R.D. (2013). Tumor cell migration in complex microenvironments. Cell Mol. Life Sci..

[73] Mondal P., Meeran S.M. (2020). Long non-coding RNAs in breast cancer metastasis. Non-Coding RNA Res..

[74] Khanh V.C., Fukushige M., Moriguchi K., Yamashita T., Osaka M., Hiramatsu Y., Ohneda O. (2020). Type 2 Diabetes Mellitus Induced Paracrine Effects on Breast Cancer Metastasis Through Extracellular Vesicles Derived from Human Mesenchymal Stem Cells. Stem Cells Dev..

[75] Du L., Tao X., Shen X. (2021). Human umbilical cord mesenchymal stem cell-derived exosomes inhibit migration and invasion of breast cancer cells via miR-21-5p/ZNF367 pathway. Breast Cancer (Tokyo Jpn.).

[76] Shojaei S., Hashemi S.M., Ghanbarian H., Sharifi K., Salehi M., Mohammadi-Yeganeh S. (2021). Delivery of miR-381-3p Mimic by Mesenchymal Stem Cell-Derived Exosomes Inhibits Triple Negative Breast Cancer Aggressiveness; an In Vitro Study. Stem Cell Rev. Rep..

[77] Egea V., Kessenbrock K., Lawson D., Bartelt A., Weber C., Ries C. (2021). Let-7f miRNA regulates SDF-1α- and hypoxia-promoted migration of mesenchymal stem cells and attenuates mammary tumor growth upon exosomal release. Cell Death Dis..

[78] Adams R.H., Alitalo K. (2007). Molecular regulation of angiogenesis and lymphangiogenesis. Nat. Rev. Mol. Cell Biol..

[79] Schneider B.P., Miller K.D. (2005). Angiogenesis of breast cancer. J. Clin. Oncol..

[80] Toffoli S., Roegiers A., Feron O., Van Steenbrugge M., Ninane N., Raes M., Michiels C. (2009). Intermittent hypoxia is an angiogenic inducer for endothelial cells: Role of HIF-1. Angiogenesis.

[81] Fox S.B., Generali D.G., Harris A.L. (2007). Breast tumour angiogenesis. Breast Cancer Res..

[82] Merino-González C., Zuñiga F.A., Escudero C., Ormazabal V., Reyes C., Nova-Lamperti E., Salomón C., Aguayo C. (2016). Mesenchymal Stem Cell-Derived Extracellular Vesicles Promote Angiogenesis: Potencial Clinical Application. Front. Physiol..

[83] Carmeliet P. (2005). VEGF as a key mediator of angiogenesis in cancer. Oncology.

[84] Melincovici C.S., Boşca A.B., Şuşman S., Mărginean M., Mihu C., Istrate M., Moldovan I.M., Roman A.L., Mihu C.M. (2018). Vascular endothelial growth factor (VEGF)—Key factor in normal and pathological angiogenesis. Rom. J. Morphol. Embryol..

[85] Lee J.K., Park S.R., Jung B.K., Jeon Y.K., Lee Y.S., Kim M.K., Kim Y.G., Jang J.Y., Kim C.W. (2013). Exosomes derived from mesenchymal stem cells suppress angiogenesis by down-regulating VEGF expression in breast cancer cells. PLoS ONE.

[86] Forsythe J.A., Jiang B.H., Iyer N.V., Agani F., Leung S.W., Koos R.D., Semenza G.L. (1996). Activation of vascular endothelial growth factor gene transcription by hypoxia-inducible factor 1. Mol. Cell Biol..

[87] Humar R., Kiefer F.N., Berns H., Resink T.J., Battegay E.J. (2002). Hypoxia enhances vascular cell proliferation and angiogenesis in vitro via rapamycin (mTOR)-dependent signaling. Faseb. J..

[88] Del Bufalo D., Ciuffreda L., Trisciuoglio D., Desideri M., Cognetti F., Zupi G., Milella M. (2006). Antiangiogenic potential of the Mammalian target of rapamycin inhibitor temsirolimus. Cancer Res..

[89] Pakravan K., Babashah S., Sadeghizadeh M., Mowla S.J., Mossahebi-Mohammadi M., Ataei F., Dana N., Javan M. (2017). MicroRNA-100 shuttled by mesenchymal stem cell-derived exosomes suppresses in vitro angiogenesis through modulating the mTOR/HIF-1α/VEGF signaling axis in breast cancer cells. Cell. Oncol. (Dordr.).

[90] Blankenstein T., Coulie P.G., Gilboa E., Jaffee E.M. (2012). The determinants of tumour immunogenicity. Nat. Rev. Cancer.

[91] Messerschmidt J.L., Prendergast G.C., Messerschmidt G.L. (2016). How Cancers Escape Immune Destruction and Mechanisms of Action for the New Significantly Active Immune Therapies: Helping Nonimmunologists Decipher Recent Advances. Oncologist.

[92] Henriksen A., Dyhl-Polk A., Chen I., Nielsen D. (2019). Checkpoint inhibitors in pancreatic cancer. Cancer Treat. Rev..

[93] Robbins P.D., Morelli A.E. (2014). Regulation of immune responses by extracellular vesicles. Nat. Rev. Immunol..

[94] Biswas S., Mandal G., Roy Chowdhury S., Purohit S., Payne K.K., Anadon C., Gupta A., Swanson P., Yu X., Conejo-Garcia J.R. (2019). Exosomes Produced by Mesenchymal Stem Cells Drive Differentiation of Myeloid Cells into Immunosuppressive M2-Polarized Macrophages in Breast Cancer. J. Immunol. (Baltim. Md. 1950).

[95] Chulpanova D.S., Gilazieva Z.E., Kletukhina S.K., Aimaletdinov A.M., Garanina E.E., James V., Rizvanov A.A., Solovyeva V.V. (2021). Cytochalasin B-Induced Membrane Vesicles from Human Mesenchymal Stem Cells Overexpressing IL2 Are Able to Stimulate CD8(+) T-Killers to Kill Human Triple Negative Breast Cancer Cells. Biology.

[96] Summers M.A., McDonald M.M., Croucher P.I. (2020). Cancer Cell Dormancy in Metastasis. Cold Spring Harb. Perspect. Med..

[97] Yeh A.C., Ramaswamy S. (2015). Mechanisms of Cancer Cell Dormancy--Another Hallmark of Cancer?. Cancer Res..

[98] Endo H., Inoue M. (2019). Dormancy in cancer. Cancer Sci..

[99] Clements M.E., Johnson R.W. (2019). Breast Cancer Dormancy in Bone. Curr. Osteoporos. Rep..

[100] Aguirre-Ghiso J.A. (2007). Models, mechanisms and clinical evidence for cancer dormancy. Nat. Rev. Cancer.

[101] Casson J., Davies O.G., Smith C.A., Dalby M.J., Berry C.C. (2018). Mesenchymal stem cell-derived extracellular vesicles may promote breast cancer cell dormancy. J. Tissue Eng..

[102] Mohd Ali N., Yeap S.K., Ho W.Y., Boo L., Ky H., Satharasinghe D.A., Tan S.W., Cheong S.K., Huang H.D., Lan K.C. (2020). Adipose MSCs Suppress MCF7 and MDA-MB-231 Breast Cancer Metastasis and EMT Pathways Leading to Dormancy via Exosomal-miRNAs Following Co-Culture Interaction. Pharmaceuticals.

[103] Ono M., Kosaka N., Tominaga N., Yoshioka Y., Takeshita F., Takahashi R.U., Yoshida M., Tsuda H., Tamura K., Ochiya T. (2014). Exosomes from bone marrow mesenchymal stem cells contain a microRNA that promotes dormancy in metastatic breast cancer cells. Sci. Signal..

[104] Bliss S.A., Sinha G., Sandiford O.A., Williams L.M., Engelberth D.J., Guiro K., Isenalumhe L.L., Greco S.J., Ayer S., Bryan M. (2016). Mesenchymal Stem Cell-Derived Exosomes.s Stimulate Cycling Quiescence and Early Breast Cancer Dormancy in Bone Marrow. Cancer Res..

[105] Sandiford O.A., Donnelly R.J., El-Far M.H., Burgmeyer L.M., Sinha G., Pamarthi S.H., Sherman L.S., Ferrer A.I., DeVore D.E., Patel S.A. (2021). Mesenchymal Stem Cell-Secreted Extracellular Vesicles Instruct Stepwise Dedifferentiation of Breast Cancer Cells into Dormancy at the Bone Marrow Perivascular Region. Cancer Res..

[106] Kuczynski E.A., Sargent D.J., Grothey A., Kerbel R.S. (2013). Drug rechallenge and treatment beyond progression--implications for drug resistance. Nat. Rev. Clin. Oncol..

[107] Mallini P., Lennard T., Kirby J., Meeson A. (2014). Epithelial-to-mesenchymal transition: What is the impact on breast cancer stem cells and drug resistance. Cancer Treat. Rev..

[108] Vasan N., Baselga J., Hyman D.M. (2019). A view on drug resistance in cancer. Nature.

[109] Zhao C.Y., Cheng R., Yang Z., Tian Z.M. (2018). Nanotechnology for Cancer Therapy Based on Chemotherapy. Molecules.

[110] Luo T., Liu Q., Tan A., Duan L., Jia Y., Nong L., Tang J., Zhou W., Xie W., Lu Y. (2020). Mesenchymal Stem Cell-Secreted Exosome Promotes Chemoresistance in Breast Cancer via Enhancing miR-21-5p-Mediated S100A6 Expression. Mol. Ther. Oncolytics.

[111] Jia Z., Zhu H., Sun H., Hua Y., Zhang G., Jiang J., Wang X. (2020). Adipose Mesenchymal Stem Cell-Derived Exosomal microRNA-1236 Reduces Resistance of Breast Cancer Cells to Cisplatin by Suppressing SLC9A1 and the Wnt/β-Catenin Signaling. Cancer Manag. Res..

[112] Maughan K.L., Lutterbie M.A., Ham P.S. (2010). Treatment of breast cancer. Am. Fam. Physician.

[113] Clarke M., Collins R., Darby S., Davies C., Elphinstone P., Evans V., Godwin J., Gray R., Hicks C., James S. (2005). Effects of radiotherapy and of differences in the extent of surgery for early breast cancer on local recurrence and 15-year survival: An overview of the randomised trials. Lancet.

[114] Arora A., Scholar E.M. (2005). Role of tyrosine kinase inhibitors in cancer therapy. J. Pharm. Exp..

[115] Drăgănescu M., Carmocan C. (2017). Hormone Therapy in Breast Cancer. Chirurgia (Bucur.).

[116] Kim S.M., Kim H.S. (2017). Engineering of extracellular vesicles as drug delivery vehicles. Stem Cell Investig..

[117] Lin Y., Lu Y., Li X. (2020). Biological characteristics of exosomes and genetically engineered exosomes for the targeted delivery of therapeutic agents. J. Drug Target..

[118] Lai R.C., Yeo R.W., Tan K.H., Lim S.K. (2013). Mesenchymal stem cell exosome ameliorates reperfusion injury through proteomic complementation. Regen. Med..

[119] Gomari H., Forouzandeh Moghadam M., Soleimani M., Ghavami M., Khodashenas S. (2019). Targeted delivery of doxorubicin to HER2 positive tumor models. Int. J. Nanomed..

[120] Melzer C., Ohe J.V., Hass R. (2020). Anti-Tumor Effects of Exosomes Derived from Drug-Incubated Permanently Growing Human MSC. Int. J. Mol. Sci..

[121] Sheykhhasan M., Kalhor N., Sheikholeslami A., Dolati M., Amini E., Fazaeli H. (2021). Exosomes of Mesenchymal Stem Cells as a Proper Vehicle for Transfecting miR-145 into the Breast Cancer Cell Line and Its Effect on Metastasis. BioMed. Res. Int..

[122] Squadrito M.L., Baer C., Burdet F., Maderna C., Gilfillan G.D., Lyle R., Ibberson M., De Palma M. (2014). Endogenous RNAs modulate microRNA sorting to exosomes and transfer to acceptor cells. Cell Rep..

[123] Xiao J., Pan Y., Li X.H., Yang X.Y., Feng Y.L., Tan H.H., Jiang L., Feng J., Yu X.Y. (2016). Cardiac progenitor cell-derived exosomes prevent cardiomyocytes apoptosis through exosomal miR-21 by targeting PDCD4. Cell Death Dis..

[124] Feng Y., Huang W., Wani M., Yu X., Ashraf M. (2014). Ischemic preconditioning potentiates the protective effect of stem cells through secretion of exosomes by targeting Mecp2 via miR-22. PLoS ONE.

[125] Vakhshiteh F., Rahmani S., Ostad S.N., Madjd Z., Dinarvand R., Atyabi F. (2021). Exosomes derived from miR-34a-overexpressing mesenchymal stem cells inhibit in vitro tumor growth: A new approach for drug delivery. Life Sci..

[126] O’Brien K.P., Khan S., Gilligan K.E., Zafar H., Lalor P., Glynn C., O’Flatharta C., Ingoldsby H., Dockery P., De Bhulbh A. (2018). Employing mesenchymal stem cells to support tumor-targeted delivery of extracellular vesicle (EV)-encapsulated microRNA-379. Oncogene.

[127] Kalimuthu S., Gangadaran P., Rajendran R.L., Zhu L., Oh J.M., Lee H.W., Gopal A., Baek S.H., Jeong S.Y., Lee S.W. (2018). A New Approach for Loading Anticancer Drugs Into Mesenchymal Stem Cell-Derived Exosome Mimetics for Cancer Therapy. Front. Pharmacol..

[128] Melzer C., Rehn V., Yang Y., Bähre H., von der Ohe J., Hass R. (2019). Taxol-Loaded MSC-Derived Exosomes Provide a Therapeutic Vehicle to Target Metastatic Breast Cancer and Other Carcinoma Cells. Cancers.

[129] Peng H., Ji W., Zhao R., Yang J., Lu Z., Li Y., Zhang X. (2020). Exosome: A significant nano-scale drug delivery carrier. J. Mater. Chem. B.

[130] Naseri Z., Oskuee R.K., Jaafari M.R., Forouzandeh Moghadam M. (2018). Exosome-mediated delivery of functionally active miRNA-142-3p inhibitor reduces tumorigenicity of breast cancer in vitro and in vivo. Int. J. Nanomed..

[131] Naseri Z., Oskuee R.K., Forouzandeh-Moghadam M., Jaafari M.R. (2020). Delivery of LNA-antimiR-142-3p by Mesenchymal Stem Cells-Derived Exosomes to Breast Cancer Stem Cells Reduces Tumorigenicity. Stem Cell Rev. Rep..

[132] Li T., Zhou X., Wang J., Liu Z., Han S., Wan L., Sun X., Chen H. (2020). Adipose-derived mesenchymal stem cells and extracellular vesicles confer antitumor activity in preclinical treatment of breast cancer. Pharmacol. Res..

[133] Swain S.M., Whaley F.S., Ewer M.S. (2003). Congestive heart failure in patients treated with doxorubicin: A retrospective analysis of three trials. Cancer.

[134] Liu M., Hu Y., Chen G. (2020). The Antitumor Effect of Gene-Engineered Exosomes in the Treatment of Brain Metastasis of Breast Cancer. Front. Oncol..

[135] Lin W., Huang L., Li Y., Fang B., Li G., Chen L., Xu L. (2019). Mesenchymal Stem Cells and Cancer: Clinical Challenges and Opportunities. Biomed. Res. Int..

[136] Vakhshiteh F., Atyabi F., Ostad S.N. (2019). Mesenchymal stem cell exosomes: A two-edged sword in cancer therapy. Int. J. Nanomed..

[137] Xunian Z., Kalluri R. (2020). Biology and therapeutic potential of mesenchymal stem cell-derived exosomes. Cancer Sci..

[138] Weng Z., Zhang B., Wu C., Yu F., Han B., Li B., Li L. (2021). Therapeutic roles of mesenchymal stem cell-derived extracellular vesicles in cancer. J. Hematol. Oncol..

[139] Torsvik A., Bjerkvig R. (2013). Mesenchymal stem cell signaling in cancer progression. Cancer Treat. Rev..

[140] Sharma A. (2018). Role of stem cell derived exosomes in tumor biology. Int. J. Cancer.

[141] Paquet J., Deschepper M., Moya A., Logeart-Avramoglou D., Boisson-Vidal C., Petite H. (2015). Oxygen Tension Regulates Human Mesenchymal Stem Cell Paracrine Functions. Stem Cells Transl. Med..

[142] Badana A., Chintala M., Varikuti G., Pudi N., Kumari S., Kappala V.R., Malla R.R. (2016). Lipid Raft Integrity Is Required for Survival of Triple Negative Breast Cancer Cells. J. Breast Cancer.

[143] Qian X.L., Pan Y.H., Huang Q.Y., Shi Y.B., Huang Q.Y., Hu Z.Z., Xiong L.X. (2019). Caveolin-1: A multifaceted driver of breast cancer progression and its application in clinical treatment. Onco. Targets.

[144] Paget S. (1889). The distribution of secondary growths in cancer of the breast. Cancer Metastasis Rev..

[145] Liu Y., Cao X. (2016). Characteristics and Significance of the Pre-metastatic Niche. Cancer Cell.

[146] Hill B.S., Sarnella A., D’Avino G., Zannetti A. (2020). Recruitment of stromal cells into tumour microenvironment promote the metastatic spread of breast cancer. Semin. Cancer Biol..

[147] Valenzuela Alvarez M., Gutierrez L.M., Correa A., Lazarowski A., Bolontrade M.F. (2019). Metastatic Niches and the Modulatory Contribution of Mesenchymal Stem Cells and Its Exosomes. Int. J. Mol. Sci..

[148] Vallabhaneni K.C., Hassler M.Y., Abraham A., Whitt J., Mo Y.Y., Atfi A., Pochampally R. (2016). Mesenchymal Stem/Stromal Cells under Stress Increase Osteosarcoma Migration and Apoptosis Resistance via Extracellular Vesicle Mediated Communication. PLoS ONE.

[149] Pasquier J., Thawadi H.A., Ghiabi P., Abu-Kaoud N., Maleki M., Guerrouahen B.S., Vidal F., Courderc B., Ferron G., Martinez A. (2014). Microparticles mediated cross-talk between tumoral and endothelial cells promote the constitution of a pro-metastatic vascular niche through Arf6 up regulation. Cancer Microenviron..

[150] Sanmartin M.C., Borzone F.R., Giorello M.B., Pacienza N., Yannarelli G., Chasseing N.A. (2021). Bone marrow/bone pre-metastatic niche for breast cancer cells colonization: The role of mesenchymal stromal cells. Crit. Rev. Oncol. Hematol..

